# Molecular dynamics simulations and in silico peptide ligand screening of the Elk-1 ETS domain

**DOI:** 10.1186/1758-2946-3-49

**Published:** 2011-11-01

**Authors:** Abrar Hussain, Peter E Shaw, Jonathan D Hirst

**Affiliations:** 1 School of Chemistry, University of Nottingham, University Park, Nottingham NG7 2RD, UK; 2 School of Biomedical Sciences, Queen's Medical Centre, Nottingham NG7 2UH, UK

## Abstract

**Background:**

The Elk-1 transcription factor is a member of a group of proteins called ternary complex factors, which serve as a paradigm for gene regulation in response to extracellular signals. Its deregulation has been linked to multiple human diseases including the development of tumours. The work herein aims to inform the design of potential peptidomimetic compounds that can inhibit the formation of the Elk-1 dimer, which is key to Elk-1 stability. We have conducted molecular dynamics simulations of the Elk-1 ETS domain followed by virtual screening.

**Results:**

We show the ETS dimerisation site undergoes conformational reorganisation at the *α*1*β*1 loop. Through exhaustive screening of di- and tri-peptide libraries against a collection of ETS domain conformations representing the dynamics of the loop, we identified a series of potential binders for the Elk-1 dimer interface. The di-peptides showed no particular preference toward the binding site; however, the tri-peptides made specific interactions with residues: Glu17, Gln18 and Arg49 that are pivotal to the dimer interface.

**Conclusions:**

We have shown molecular dynamics simulations can be combined with virtual peptide screening to obtain an exhaustive docking protocol that incorporates dynamic fluctuations in a receptor. Based on our findings, we suggest experimental binding studies to be performed on the 12 SILE ranked tri-peptides as possible compounds for the design of inhibitors of Elk-1 dimerisation. It would also be reasonable to consider the score-ranked tri-peptides as a comparative test to establish whether peptide size is a determinant factor of binding to the ETS domain.

## Background

Regulation of gene expression is essential for the development of all living organisms through processes such as cell proliferation, differentiation and morphogenesis. Key to these processes are mitogen activated protein kinases (MAPK), which target nuclear transcription factors, in response to extracellular signals, to elicit the required genetic response. One such transcription factor is Elk-1. Elk-1 (Ets-like protein 1) is a member of a group of proteins called ternary complex factors (TCF), which are targeted by MAPKs for phosphorylation [1-3] to regulate the transcription of immediate early genes (IEG) [4,5]. This event involves the formation of a ternary complex, induced by the cooperative binding of TCFs with serum response factor (SRF) dimers [6] on serum response elements found in IEG promoters [7-9]. TCFs are a subfamily of ETS (Etwenty-six) domain proteins. ETS proteins share a ~85 residue DNA-binding domain (ETS domain) located at the N-terminus of TCFs, which comprises a 'winged helix-turn-helix' motif [10] that binds to a 10-bp ETS binding site containing a 5*'*-GGA-3*' *core sequence. Since ETS domains are highly conserved across ETS proteins, ETS binding sites are differentiated by the cooperation of other transcription factors [7,11,12] combined with base-specific interaction with variable bases flanking the central core sequence. Whilst TCFs naturally form a complex with SRF, they are also able to bind to DNA containing high-affinity, autonomous ETS binding motifs independent of a SRF [6,13]. ETS domain proteins are involved in cellular development, growth and differentiation [14-16]. Their deregulation has been linked to multiple human diseases [17].

**Figure 1:**
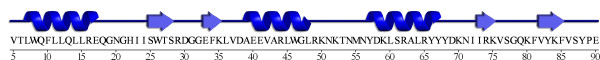
**ETS domain secondary structure**. Amino acid sequence of the Elk-1 ETS domain, showing the locations of *α*-helices and *β*-strands.

**Figure 2:**
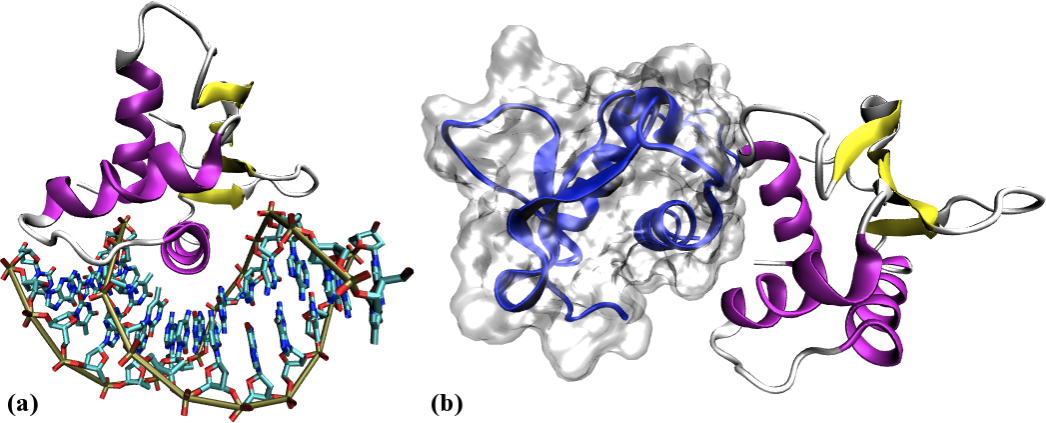
**ETS domain DNA and dimer complexes**. (a) An Elk-1 ETS domain bound to its DNA recognition sequence. *α*-helices are in purple and *β*-strands in yellow. (b) An Elk-1 ETS domain dimer complex, showing the *α*1*β*1 loops providing the interface. The images were generated using VMD [63] and PovRay (http://www.povray.org/), using coordinates taken from the 1DUX crystal structure [18].

The current X-ray crystal structure of the Elk-1 ETS domain is that of a dimer, with each unit bound to an autonomous 13-bp DNA double helix (PDB code 1DUX) [18] composed of a high affinity ETS binding site motif. Like other ETS domain proteins, the structure reveals three *α*-helices packed against four anti-parallel *β*-strands, giving an *αββααββ *secondary structure (Figure 1). The *α*3 helix forms the recognition helix, which slots into the major groove of the DNA target with a GGA core (Figure 2a). The dimer interface involves the carboxy-end of *α*1 and the *α*1*β*1 loop (Figure 2b). Contrary to the aforementioned structure, unequivocal experimental evidence has indicated that ETS dimers exist only in solution, [19,20] whilst monomers occur predominantly in the nucleus, where they target DNA [21,22]. To date, the structure of an unbound ETS domain is yet to be reported. However, Saven *et al*. [23] performed molecular dynamics (MD) simulations of a single Elk-1 ETS domain taken from the dimeric structure. They discerned regions within the simulated monomeric structure which showed large structural deviation with respect to the structure of the domain in the dimeric conformation. These regions include residues at the *α*1*β*1 loop involved in the ETS dimer interface and residues at the *α*2*α*3 loop involved in protein-DNA contacts.

Thus far, work on characterising the mechanism for protein-DNA recognition in TCFs has been abundant [18,23-27]. However, there has been little on understanding the basis of Elk-1 dimerisation for transcriptional activity. Shaw and colleagues [21] have identified a region of the Elk-1 ETS domain encompassing the *α*1*β*1 loop which distinctly contributes to Elk-1 stability in the cytoplasm by directing Elk-1 dimer formation. Also, dimerisation in the cytoplasm appears to prevent rapid degradation and plays a role in translocation of the protein to the nucleus and its subsequent accumulation therein.

In the current work, we identify a series of peptides that can serve as leads for the design of potential peptidomimetic inhibitors of Elk-1 dimerisation. Using a docking-based approach, we screened entire libraries of all possible di- and tri-peptides against the Elk-1 ETS domain, targeting the stability region of the domain identified by Shaw *et al *[21]. Given the findings of Saven *et al*., [23] it was essential to consider possible structural deviations or fluctuations in the *α*1*β*1 loop region that may affect binding of such inhibitors. Therefore, we performed MD simulations for an Elk-1 ETS monomer, to generate an ensemble of monomeric ETS conformations to use as docking targets. Herein, we show that tri-peptides appear to be good candidates for the design of inhibitors/binders of the Elk-1 dimer interface, based on size and binding specificity; di-peptides, on the other hand, appeared to behave as generic protein surface binders. We have also identified a set of tri-peptides, which may bind competitively to the ETS dimer interface.

## Computational Methods

### Molecular Dynamics Simulations

All stages of the MD simulations were carried out using CHARMM version 34b1 [28,29] with the all-atom CHARMM22 force field [30] and CMAP extensions [31-33]. Our initial structure of a representative ETS domain monomer was chain C from the 1DUX crystal structure [18]. For residues with alternative positions, the pose with the highest occupancy was retained. Hydrogen atoms were assigned using the HBUILD module [34]. The system underwent three rounds of energy minimisation using the conjugated gradient method to remove any unphysical contacts until the system had converged. During the minimization all non-hydrogen atoms were harmonically restrained with a force constant of 30 kcal mol^-1 ^Å^-1^, which was reduced by 10 kcal mol^-1 ^Å^-1 ^at each successive round. The system was solvated in a cubic solvation box (62.2 Å × 62.2 Å × 62.2 Å), containing 7460 TIP3P water molecules, [35] using periodic boundary conditions. The fully solvated system was minimised using the conjugated gradient method. First, the protein was fixed to allow the water molecules to minimise and then harmonically restrained with a force constant of 30 kcal mol^-1 ^Å^-1^. A switched cut-off was used at an atom-pair distance of 10 Å for calculations of non-bonded interactions with a 2.0 Å switching region. The Particle Mesh Ewald algorithm was used for calculating long-range electrostatic interactions [36]. The system was gradually heated from 0 K to 300 K and allowed to equilibrate for 100 ps. The SHAKE algorithm [37] was applied to constrain all hydrogen-heavy atom bonds to remove the need to sample the high frequency vibrations. Simulations were performed with a 1 fs timestep with the Leapfrog integrator. Following equilibration, the simulation continued for a further 4 ns in the isobaric-isothermal (constant pressure and temperature, NPT) ensemble for the production run. During this phase, structural coordinates of the system were taken at 0.1 ps intervals to build a trajectory of the system dynamics. Time-dependent properties were calculated from the production trajectory. In preparation for this, the C*_α _*atoms from each frame of the trajectory were aligned using least-squares fitting to the coordinates of the starting conformation. The root mean square deviation (RMSD) from the initial conformation and radius of gyration were calculated to survey any structural fluctuations over the time-series. To evaluate local structural deviations between the simulated ETS monomer conformations and the initial dimmer conformation, a residue-specific RMSD of main-chain atoms (N, C*_α_*, C, O) was calculated, averaged for the entire conformational ensemble. To complement this, we examined the changes in the backbone dihedral angles for structural fluctuations at residues around the *α*1*β*1 loop region (16-23).

Several snapshots were extracted from the trajectory to represent the various conformations for an Elk-1 ETS monomer. This was done by clustering the trajectory using backbone dihedral angles for residues 20-22 and selecting the conformation closest to the centre of each cluster as a representative conformation. The threshold defining the size of each cluster was the average of the standard deviation for the six chosen angles, over the time-series.

### Automated Peptide Docking

Libraries of all possible di-and tri-peptide were built using all 20 standard, genetically encoded amino acids (400 di-peptides and 8,000 tri-peptides). The first step was to generate a SMILES string [38] from the raw peptide sequences, using ChemAxon's MolConverter program [39]. For each peptide, tautomers at physiological pH (7.4) were produced using ChemAxon's Calculator Plugins [40]. Any unreasonable peptide structures were removed from each library, including any structures with protonated carbonyl groups, de-protonated amines, structures without formally charged termini, and structures with anionic amides. Each peptide library was docked to the ETS monomer conformations obtained from the clustering. The dockings were carried out using OpenEye's docking program FRED, [41] a rigid docking algorithm, which requires a pre-computed conformer ensemble for screening the conformational space of the ligands. The conformer ensembles were created using Omega version 2.3.2 (OpenEye Scientific Software) [42]. A maximum of 500 low energy conformers were constructed for each peptide, *in vacuo*, using the MMFF94s force field [43,44]. The Coulombic and attractive part of the van der Waals terms were excluded from the force field, to reduce the effects of strong intermolecular interactions (e.g. hydrogen bonds) that can result in folded (peptide) conformations. Conformers with an energy difference greater than 25 kcal mol^-1 ^from the lowest energy conformer were rejected and conformers in the final ensemble were required to have a heavy atom RMSD greater than the duplicate removal threshold (0.4 Å). These settings were in line with the "high quality screening" settings of Kirchmair *et al *[45]. All remaining parameters were the default values.

The docking site for each receptor was delineated by a grid box encasing residues at the Elk-1 dimer interface site. A protein contact constraint, which all successful dockings were require to satisfy, was defined on Leu45, which is a key pharmacophoric contact for the dimer interface. The di- and tri-peptide libraries (with conformers) were separately docked, using FRED version 2.2.5, to each of the Elk-1 ETS domain conformations. Each multi-conformer peptide-ligand was exhaustively docked to a receptor using default step-sizes and the ChemGauss2 scoring function (a propriety function of OpenEye) [41].

ChemGauss2 is a chemically aware shape-fitting scoring function, which uses Gaussian functions to describe the shape and chemistry of molecules. The best scoring poses for each compound were optimised in their docked state by half a rotation and translation step in each direction using the OEChemScore scoring function. OEChemscore is an OpenEye variant of the Chemscore [46] scoring function, but lacks a component for an entropy penalty upon complex formation.

On completion of the docking simulations, the single highest-scoring tautomeric state of each peptide was taken to give 400 unique di-peptides and 8,000 unique tri-peptides. Results from both libraries were analysed similarly but separately. The peptides were initially ranked by docking score, where rank 1 corresponded to the highest scoring peptide. Due to the variability in the size of peptides in both libraries, where size is a simple heavy atom count (HAC), and a systematic bias in the scoring functions (including OEChemscore), [47,48] we employed a simple size-independent metric to rank peptides and select the best binders. We used the size-independent ligand efficiency (SILE) metric [49]:

(1) $$SILE\;=\frac{affinity}{HAC^{1-x}}$$

where *affinity *can be any binding measurement, in our case the docking score; *x *is derived by fitting the maximal ligand efficiency (LE*_max_*) values from all 12 docking screens against HAC, to a logarithmic function of the form:

(2) $$\text{ln}\left(LE_{max}\right)=k-x\;\text{ln}\left(HAC\right)$$

Docking data from the di- and tri-peptide sets were fitted and examined separately.

Docked complexes between the highest-ranked peptides and the 12 protein conformations were analysed using HBPLUS, using default parameters [50]. Only hydrogen bonds between protein and peptide ligands were considered. The number of ETS residues participating in interactions with the top SILE-ranked peptide in each complex was counted. This count was also dissected into the number of specific contacts made, where specificity is defined as interactions between peptide side-chains and ETS residues.

## Results and Discussion

### Analysis of Elk-1 dimer interface

**Figure 3:**
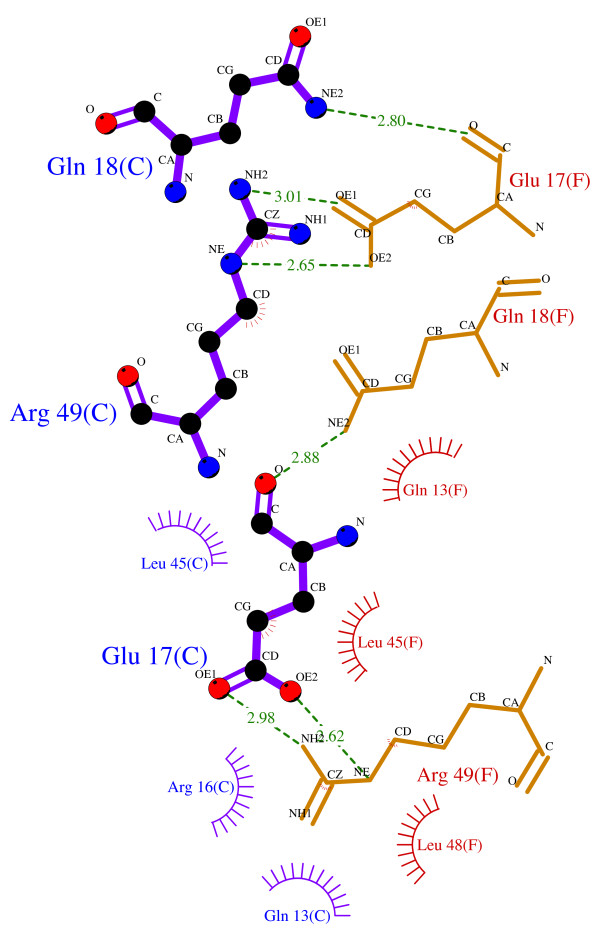
**ETS domain dimer interface**. LIGPLOT representation of intermolecular interactions between two Elk-1 ETS domains according to the X-ray crystal structure (1DUX) of the dimer complex. Non-bonded interactions are indicated by spokes and hydrogen bonds by dashed green lines, with lengths given in Å. Residues from chain C are shown with purple bonds and chain F in orange.

In order to aid the identification of possible peptide binders for the Elk-1 dimer interface, it was important to identify structural features contributing the dimerisation. Interactions between two Elk-1 ETS domains were calculated using the LIGPLOT program [51]. The minimum and maximum interatomic bond distances for non-bonded contacts were 2.90 Å and 3.90 Å, respectively, and for hydrogen bonds: 2.70 Å and 3.35 Å. The LIGPLOT diagram for chains C and F from the X-ray crystal structure of the ETS dimer (Figure 3) reveals a homodimeric interaction between the two ETS domains. Key to the interface were residues 17, 18 and 49, where Gln18 and Arg49 of one domain donate three hydrogen bonds to Glu17 of the partnering domain. Accompanying these hydrogen bond interactions, several residues make large steric contributions to the interface; these are listed in Table 1 together with a percentage accessible surface area of the interface, calculated using NACCESS [52]. The schematic depicting the secondary structure of the ETS domain in Figure 1 shows the relative positions of these residues in the domain.

**Table 1:** Residue contribution to the dimer interface accessible surface area (ASA), calculated using NACCESS [52]

Residue	% contribution to interface ASA
Gln13	4.6
Arg16	17.6
Glu17	22.0
Gln18	8.8
Gly19	2.6
Asn20	5.3
Leu45	8.5
Leu48	8.4
Arg49	14.4

### MD simulations of an Elk-1 ETS domain

**Figure 4:**
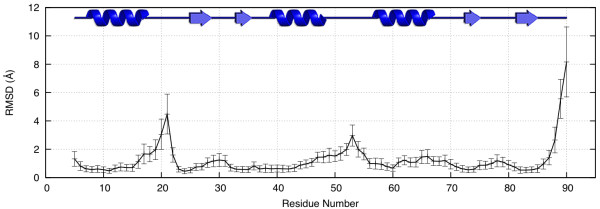
**Residue specific ETS monomer fluctuations**. Time-averaged RMSD for the main-chain atoms of each residue over 4 ns of simulation of an Elk-1 ETS domain. The bars signify fluctuations about the mean and correspond to one standard deviation.

Over the course of the MD simulation, the radius of gyration (RoG) and the RMSD of the backbone atoms relative to the minimised (initial) structure of each frame in the trajectory remained stable. The mean values for the RMSD and the RoG were 1.64 ± 0.24 Å and 12.17 ± 0.08 Å, respectively. The latter was, in fact, identical to the RoG of the initial structure. This indicated that the overall shape and size (packing) of both the monomeric and dimeric conformation of the Elk-1 ETS domain is conserved. To focus on localised structural deviations, we calculated the time-averaged RMSD for each residue, with respect to the main-chain atoms of the initial conformation. This revealed substantial structural deviations for residues 20-22 compared to the dimeric conformation (Figure 4). These residues are situated at the centre of the *α*1*β*1 loop, which was identified by Shaw *et al*. [21] as the region accountable for Elk-1 stability. We also measured the backbone dihedral angles for residues in the loop across the entire trajectory. Residues 16 to 19 and residue 23 showed dihedral angle fluctuations within range of typical thermal fluctuations for proteins, with an average standard deviation about the mean of ±19° across the trajectory for the 10 angles; fluctuations of the backbone dihedrals for residues 21 and 22 were considerably larger, with the lowest standard deviation value of ±59° and the highest of ±88°. The high fluctuation of residues 21 and 22 are consistent with the high RMSD values seen in Figure 4.

Since the structure fluctuates in the region coinciding with the *α*1*β*1 loop, which was our proposed docking binding site, it would have been unreasonable to dock to the single domain conformation taken from the crystal structure of the dimer, or to dock to the averaged structure of the MD trajectory. Instead, we clustered the trajectory, based on the backbone dihedral angles of residues 20-22, to extract several conformations representative of an Elk-1 ETS domain monomer. Using a clustering threshold of 49.2°, which was the average of the standard deviations of the six angles, 12 clusters were obtained. From each cluster, a single conformation was taken (Table 2) and used for the docking study. (see Additional file 1 for an alignment of the minimised structure and the 12 conformations).

**Table 2:** ETS monomer conformations obtained by clustering the MD trajectory on the backbone dihedral angles of residues 20-22 (cluster threshold = 49.2°)

Cluster	% of MD trajectory
ETS1	11.5
ETS2	4.0
ETS3	5.9
ETS4	8.9
ETS5	4.8
ETS6	16.4
ETS7	7.1
ETS8	1.4
ETS9	4.1
ETS10	4.6
ETS11	21.2
ETS12	10.1

### Peptide Docking

#### Peptide screening

Libraries of all possible di- and tri-peptides, together with possible tautomers of each peptide were constructed. The final libraries (including protonation and tautomeric states) were made up of 1,128 di-peptides and 33,367 tri-peptides. The two peptide libraries were individually screened against the 12 monomer conformations of the Elk-1 ETS domain. Each multi-conformer peptide-ligand was exhaustively docked to the receptors, i.e., all rigid-body translations and rotations of a conformer were enumerated within the docking site, centred on residues for the Elk-1 dimer interface. Although with different affinities, all peptides bound to the docking site with favourable scores.

**Figure 5:**
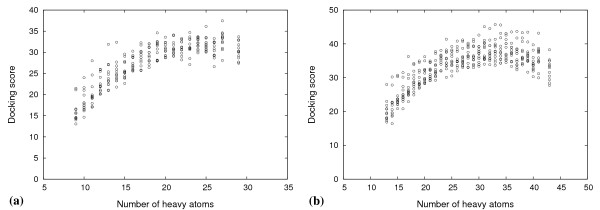
**Highest scoring peptides by size**. Highest scoring (a) di-peptides and (b) tri-peptides taken at each HAC from all 12 docking screens. Docking scores have been plotted as non-negative values for convenience.

The docked peptide-ligands for each library were ranked according to docking score, retaining only the highest scoring tautomer of each peptide. Using this simple ranking scheme, particularly for the di-peptides, peptide-ligands with a larger heavy atom count (HAC) were ranked higher than those with a smaller one. Although this effect is apparent in experimental ligand binding data, [53] unfortunately it is unduly amplified in computational docking studies. The problem stems from the additive nature of scoring functions. The scoring function tends to favour larger ligands, as they contribute towards a greater number of intermolecular interactions with the target. This phenomenon is inherent to several docking scoring functions, including OEChemscore, which lack other terms in the function, such as a desolvation penalty term, that can counter-balance the favoured interaction terms. For our peptide ligands, the effect is seen in Figures 5a and 5b for the highest scoring di- and tri-peptides, respectively, taken at each HAC from the 12 docking screens.

**Figure 6:**
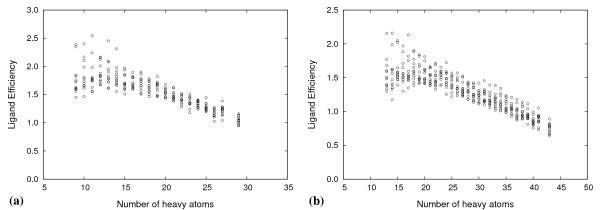
**Peptide binding efficiencies by size**. Highest ligand efficiency values for (a) di-peptides and (b) tri-peptides taken at each HAC from all 12 docking screens.

Because the bias towards larger ligands is counter to the rules for drug bioavailability, [54] a simple metric, called ligand efficiency has been developed to assess the binding of a compound, with respect to the number of atoms, and its potential for lead optimisation [53,55]. Ligand efficiency (LE) is the binding affinity (potency) divided by a measure of the size of a ligand, often the HAC, as defined by Kuntz *et al *[53]. Compounds that can provide the desired binding affinity with fewer atoms are considered efficient. However, in large screening studies of ligands spanning a wide range of molecular sizes, ligand efficiency is non-linearly related to HAC, and appears to fall as size increases [56,57]. This trend can be illustrated by plotting the LE versus HAC (Figure 6), for the peptides used in Figure 5. The trend may be related to the increased complexity of larger compounds. More complex compounds can bind a target with a less than optimal geometry, due to binding constraints and structural compromises [58]. They also offer a smaller surface area per atom to make favourable interactions compared to smaller, less complex compounds [56]. LE over-corrects for the size dependence in docking scores. Therefore, a size-normalised efficiency scale was needed. We used the size-independent ligand efficiency (SILE) [49] scale to rank peptides in the docked libraries. In order to apply a SILE metric for our data, a value for *x*, for Equation (1), was obtained by fitting the maximal LE (LE*_max_*) values taken from Figure 6 to the function in Equation (2) (see Additional file 1). LE*_max _*is the highest LE value at each HAC. The *x *values for di- and tri-peptides were 0.649 and 0.665, respectively, which were close to the generic value of 0.7 suggested by Nissink [49].

**Figure 7:**
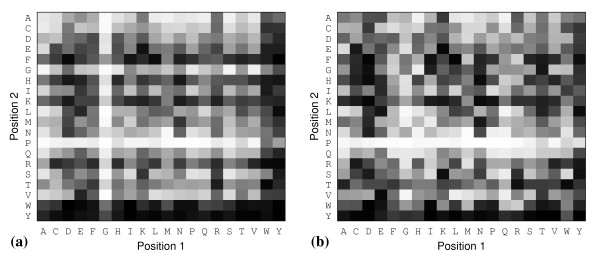
**Docked di-peptide ranking maps**. 2D-maps representing the peptide rank by (a) docking score and (b) SILE values according to the positions occupied by each residue for di-peptides docked to ETS conformation 9 (ETS9). The ranks are represented as squares shaded from black (highest rank) to white (lowest rank).

**Figure 8:**
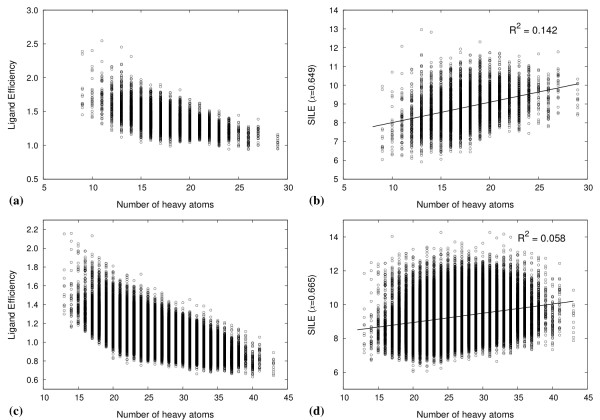
**Peptide efficiency distribution**. Distribution of LE ((a) and (c)) and SILE ((b) and (d)) values for docked di- and tri-peptides as a function of the number of heavy atoms. (a) LE and (b) SILE values for di-peptides; (c) LE and (d) SILE values for tri-peptides.

By mapping the two sequence positions for ranked di-peptides on to a 20 × 20 matrix, where each square is graded according to the associated rank, we can see the difference in size-dependence between score- and SILE-ranked results (Figures 7a and 7b). Score-ranked matrices clearly show peptides consisting of heavier amino acid residues such as tryptophan and tyrosine ranked higher, whilst those of smaller residues such as alanine and glycine ranked lower. The SILE-ranked matrices reduce this bias (Figure 7b). Similarly, plots of the distribution of LE and SILE values for the di- and tri-peptide dockings as a function of HAC reveal a reduced size-dependency for SILE values compared to LE values (compare Figure 8b with 8a and 8d with 8c). However, the SILE values for di-peptides maintain some size dependence (Figure 8b) compared to the tri-peptides (Figure 8d). It may be that the binding site readily accommodates the di-peptides, due to their smaller size and low structural complexity, and thus the size bias remains dominant. Therefore, di-peptides with a lower HAC bind and fit the binding site more completely, where a greater number of atoms participate equally in protein-peptide interactions compared to tri-peptides and di-peptides with a higher HAC. A similar result was observed in a peptide docking study to the Fv fragment of a monoclonal IgM cryoglobulin [59]. In that study, docking results were skewed towards di-peptides composed of larger residues. It was suggested that the di-peptides were too small to discriminate between different binding cavities, which is consistent to the hypothesis of 'a small ball in a large hole'. Thus, the size-independent metric is less effective for compounds of lower complexity. This also suggests that di-peptides are fairly promiscuous protein surface binders and may not offer a specific binding preference for the dimer interface site had the docking site definition been larger.

Tables 3 and 4 list the highest score- and SILE-ranked di- and tri-peptides, respectively. These tables again reveal the preference for peptides consisting of large aromatic residues for the score-ranked results, particularly for the di-peptides. In addition to the factors discussed above, it is possible such residues may behave as anchors to aid the binding of the complete peptides. However, given that a minority (38%) of residues in the dimer interface are non-polar, it is perhaps unlikely that these hydrophobic residues, especially tryptophan and tyrosine, would show particular affinity for the binding site in experimental assays.

**Table 3:** Highest score- and SILE-ranked di-peptides

	Score-ranked		SILE-ranked	
Protein conformation	Sequence	Heavy atom count	Sequence	Heavy atom count
ETS1	WY	27	YQ	22
ETS2	GK	14	GD	13
ETS3	WY	27	YG	17
ETS4	WY	27	WY	27
ETS5	TY	20	AY	18
ETS6	DW	23	YA	18
ETS7	YW	27	QY	22
ETS8	WY	27	YG	17
ETS9	YY	25	VY	20
ETS10	KE	19	KE	19
ETS11	EW	24	SY	19
ETS12	YW	27	YW	27

**Table 4:** Highest score- and SILE-ranked tri-peptides

	Score-ranked		SILE-ranked	
Protein conformation	Sequence	Heavy atom count	Sequence	Heavy atom count
ETS1	IYW	35	TKT	24
ETS2	GYY	29	GGD	17
ETS3	YKE	31	YKE	31
ETS4	KWR	35	SYG	23
ETS5	HAY	28	TTG	19
ETS6	KEY	31	KEY	31
ETS7	RYW	38	SFG	22
ETS8	LWY	35	SYA	24
ETS9	EHW	34	TDY	28
ETS10	WYV	34	KSD	24
ETS11	DYW	35	YSY	31
ETS12	KEW	33	TYF	31

#### Structural analysis of docked complexes

**Figure 9:**
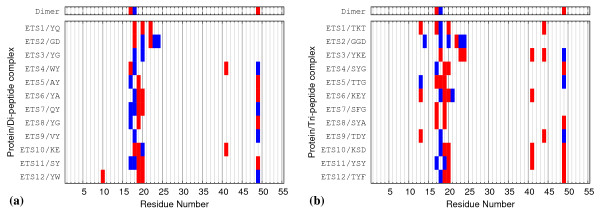
**Peptide hydrogen bond fingerprints**. Hydrogen bond "Interaction fingerprints" for docked complexes between Elk-1 ETS domain conformations and highest SILE-ranked (a) di- and (b) tri-peptides. Specific contacts, as described in the main text, are given in red and peptide main-chain contacts in blue.

Interactions between the top SILE-ranked peptide-protein complexes were calculated for each Elk-1 ETS domain conformation. Given the systematic bias in the score-ranked results, they were not considered for interaction analysis. Overall, both sets of peptides interact with residues in the dimer interface, namely the regions at sequence positions 10-20 and 40-50. To investigate the specificity of binding, the number of ETS domain residues hydrogen bonded with a peptide were counted for each of the docked complexes. On average, di-peptide ligands interacted with fewer ETS domain residues compared to tri-peptides (column 2, Tables 5 and 6), although some of these interactions did include those made to ETS domain residues Glu17, Gln18 and Arg49, which were identified as key hydrogen-bond contacts at the dimer interface (see Figure 3). In addition, the highest SILE-ranked tri-peptides make more specific contacts to the protein compared to the highest-ranked di-peptides (column 3, Tables 5 and 6). Here, we measure specificity as interactions between peptide side-chains and ETS residues. Figure 9 shows an "Interaction fingerprint" of the hydrogen bonds between the highest SILE-ranked peptide and the corresponding ETS conformations. The Elk-1 ETS domain dimer fingerprint is given at the top of the figure as a reference.

**Table 5:** Number of ETS residues participating in hydrogen bond interactions with highest SILE-ranked di-peptides

Di-peptide complex	ETS residues in peptide-protein hydrogen bonds	ETS residues in peptide specific hydrogen bonds
ETS1/YQ	3	3
ETS2/GD	5	1
ETS3/YG	2	0
ETS4/WY	4	2
ETS5/AY	3	2
ETS6/YA	4	3
ETS7/QY	5	2
ETS8/YG	3	2
ETS9/VY	2	0
ETS10/KE	4	3
ETS11/SY	5	3
ETS12/YW	4	3

**Table 6:** Number of ETS residues participating in hydrogen bond interactions with highest SILE-ranked tri-peptides

Tri-peptide complex	ETS residues in peptide-protein hydrogen bonds	ETS residues in peptide specific hydrogen bonds
ETS1/TKT	5	4
ETS2/GGD	6	1
ETS3/YKE	6	5
ETS4/SYG	4	3
ETS5/TTG	5	3
ETS6/KEY	6	4
ETS7/SFG	2	2
ETS8/SYA	3	3
ETS9/TDY	4	2
ETS10/KSD	5	4
ETS11/YSY	5	3
ETS12/TYF	4	3

Perhaps naively we may have expected a peptide corresponding to a contiguous sequence of residues involved in the Elk-1 dimer interface would have been ranked high in the docking simulation, but this was not the case. The most obvious such peptides were, the tri-peptide Arg-Glu-Gln, which corresponds to residues 16-18 in the ETS domain, and the di-peptide Glu-Gln corresponding to residues 17-18 (as seen in Figure 3). The best SILE-ranked Glu-Gln di-peptide was ranked 52 out of 400 in complex with ETS7 and had an average ranking of 133 for all 12 docked complexes. Whilst the best SILE-ranked Arg-Glu-Gln tri-peptide was ranked 1423 out of 8000 in complex with ETS8 and an average ranking of 3729 (Table 7). This is largely because these peptides, although capable of providing some of the hydrogen bond interactions found at the dimer interface, are unable to mimic the interactions of other residues involved in dimer interface (see Table 1 and Figure 3), particularly van der Waals contacts. This has been recognised in other efforts to discover small molecules that disrupt protein-protein interactions [60]. For this reason, the binding of the two aforementioned peptides may be weaker than the higher ranked peptides, which satisfy more of the pharmacophoric constraints of the dimer.

**Table 7:** SILE-rank of Glu-Gln di-peptide and Arg-Glu-Gln tri-peptide in complex with each ETS conformation

ETS conformation	Glu-Gln ranking	Arg-Glu-Gln ranking
ETS1	145	6777
ETS2	112	4685
ETS3	52	1925
ETS4	211	7690
ETS5	108	1586
ETS6	184	2292
ETS7	58	3721
ETS8	67	1423
ETS9	186	5607
ETS10	128	3378
ETS11	193	2527
ETS12	157	3134

## Conclusions

It is well-established that TCFs, such as Elk-1, play a critical role in transcriptional activation in response to extracellular signals and a consequent role in the growth and development of cells. Using MD simulations we have identified possible conformations for an Elk-1 ETS domain monomer and observed a structural variation from the dimeric form at the *α*1*β*1 loop, where two Elk-1 proteins dimerise. Against these monomeric conformations we screened all possible di- and tri-peptides and have identified several peptides with potential to mimic and possibly inhibit Elk-1 dimerisation. The size and binding specificity of the tri-peptides make them ideal candidates for the design of peptidomimetics of the Elk-1 dimer interface. The di-peptides, on the other hand, appear to be a generic set of protein surface binders and are unlikely to produce experimental binding affinity for the ETS dimer interface site that would correlate with the docking data. The notion of using tri-peptides as potential candidates for peptidomimetic design has also been supported in a recent review by Ung and Winkler [61].

Since docking scoring functions are based on a number of simplifications and assumptions, their predictions for binding free energies for a protein-ligand complex are not quantitative. This also makes it very difficult to discriminate between strong/weak binders and non-binders, particularly for a relatively at and exposed binding site, as investigated here. Although this is a major limitation in a docking protocol, the exhaustive search algorithm of docking programs has been successful in predicting correct binding geometries of known hits [48]. As with all docking protocols, true validation can only be achieved through experimental binding measurements correlating with the docking results. For an experimental binding study, it would be reasonable to test the binding affinity of the top SILE-ranked tri-peptides listed in Table 4. The score-ranked tri-peptides may also be worth considering as a comparative test to establish whether size of the peptide is a determinant factor of binding to the ETS domain or if it is, indeed, just an artefact of docking. Binding data for the Arg-Glu-Gln peptide may also be useful in explaining the poor predicted binding by the docking simulations.

It is quite clear that complex formation of a protein and ligand is a dynamic mechanism. Here we have shown a combination of MD and docking simulations can be used to provide an understanding of the effects on ligand binding to a dynamic representation of the receptor, which a single configuration crystal structure would fail to reveal. Thus, computer simulations on protein-ligand complexes can enhance crystal structure data in this respect. We plan to extend the current work by performing all-atom MD simulations of selected peptides complexed with an Elk-1 ETS domain to assess the stability of the complexes, whilst incorporating any induced fitting of the peptides and obtain accurate binding data for use in designing future docking studies of optimised peptides. We also plan to apply free energy perturbation methods [62] to a set of the best peptides to calculate relative binding free energy of alchemic transformations of the peptides in complex with the Elk-1 ETS domain. This may also go as far as identifying tetra-peptides with potentially superior binding affinities compared to the tri-peptides we have considered here.

## Competing interests

The authors declare that they have no competing interests.

## Authors' contributions

PES and JDH together conceived the study. AH and JDH devised the strategy and analyses undertaken. AH performed the calculations, analysis and drafted the manuscript. JDH supervised the study and with PES participated in the discussion of the results. All authors have read and approved the final manuscript.

## Supplementary Material

Additional file 1 **Superposition of ETS target structures and derivation of maximal ligand efficiency**. Document contains: 1) figures showing alignment of the 12 ETS target structures with the minimised structure and 2) plots showing the maximal ligand efficiency values for the docked di- and tri-peptides. Click here for file
