# Supplementary material for: Molecular dynamics simulations and in silico peptide ligand screening of the Elk-1 ETS domain
Source: J Cheminform. 2011 Nov 1;3:49. doi: 10.1186/1758-2946-3-49 (PMC3227621; doi:10.1186/1758-2946-3-49)
Supplement: Additional file 1 — Superposition of ETS target structures and derivation of maximal ligand efficiency. Document contains: 1) figures showing alignment of the 12 ETS target structures with the minimised structure and 2) plots showing the maximal ligand efficiency values for the docked di- and tri-peptides. [file 1758-2946-3-49-S1.PDF]

Molecular dynamics simulations and in silico peptide ligand screening of the Elk-1 ETS domain

Abrar Hussain[1], Peter E. Shaw[2], and Jonathan D. Hirst[1]

[1] *School of Chemistry, University of Nottingham, University Park, Nottingham NG7 2RD, UK*
[2] *School of Biomedical Sciences, Queen's Medical Centre, Nottingham NG7 2RD, UK*

Supplementary Data

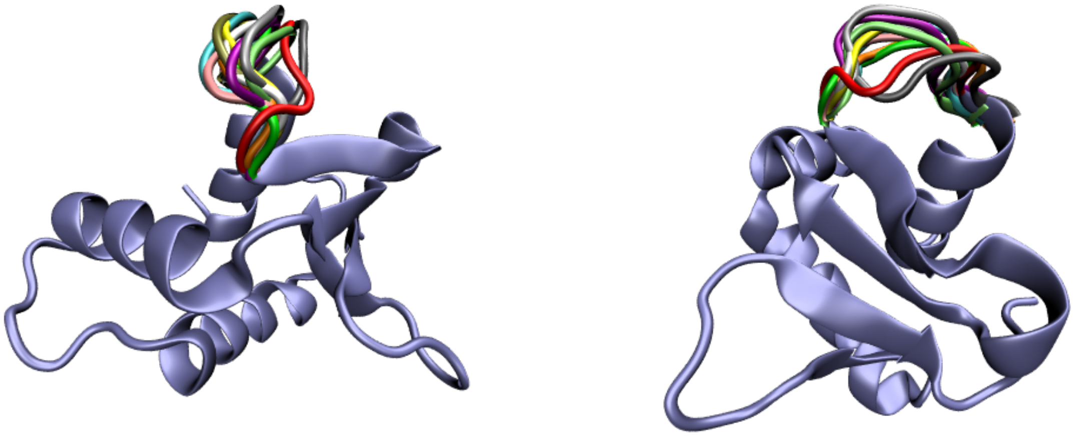

(a) (b)

Superposition of the $\alpha1\beta1$ loop region of the 12 ETS target structures and the minimised ETS domain (*blue*) according to a least-square fitting of the C_α atoms. (a) is a perspective looking directly down the loop (b) shows a perspective looking past the $\beta1\beta2\beta3$ sheet.

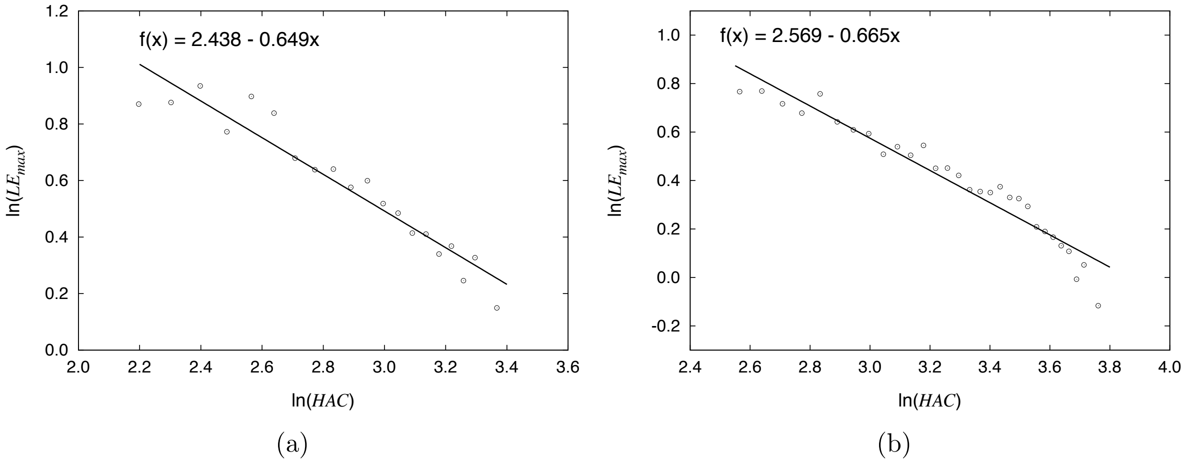

Maximal ligand efficiency values as a function of number of heavy atoms for (a) docked di-peptides and (b) docked tri-peptides from all 12 docking screens.
